# Effect of Caloric Intake 25 or 30 kcal/kg/day on the Glycemic Control in Obese Patients With Type 2 Diabetes

**DOI:** 10.4021/jocmr1488w

**Published:** 2013-08-05

**Authors:** Kiyomi Masuda, Kazutaka Aoki, Junko Kawaguchi, Tadashi Yamakawa, Ikuro Matsuba, Yasuo Terauchi

**Affiliations:** a Department of Endocrinology and Metabolism Yokohama City University Graduate School of Medicine, 3-9 Fukuura, Kanazawa-ku, Yokohama 236-0004, Japan; b Department of Endocrinology and Diabetes, Yokohama City University Medical Center, 4-57 Urafune-cho, Minami-ku, Yokohama, Kanagawa 232-0024, Japan; c The Study Group of the Diabetes Committee, Kanagawa Physicians Association, 3F Kanagawa-ken Sogo Iryo Kaikan, 3-1 Fujimi-cho, Naka-ku, Yokohama, 231-0037, Japan

**Keywords:** Diabetes mellitus, Obesity therapy, Nutrition, Energy regulation

## Abstract

**Background:**

The recommended total dietary energy intake prescribed medical nutrition therapy for obese or overweight patients with type 2 diabetes in Japan is often set at 25 kcal/kg ideal body weight (IBW)/day. This study was conducted to determine the impact of the total dietary energy intake (25 or 30 kcal/kg IBW/day) on the glycemic control, lipid profile, and satisfaction level in overweight patients with type 2 diabetes.

**Methods:**

We performed interview and a designed prospective, randomized, controlled, multicenter study trial. Recruitment for interview for doctors and hospitalization of the obese or overweight patients with type 2 diabetes began from September 2008 and continued until June 2010. The subjects were randomly assigned to 25 kcal/kg IBW/day group (25 kcal group) or 30 kcal/kg IBW/day group (30 kcal group). The primary endpoint was the body weight of the subjects at the time of hospitalization, at the time of discharge from the hospital, and at 3, 6 and 12 months after discharge from the hospital.

**Results:**

The glycemic control, lipid control and body weight were similar between the 25 and 30 kcal groups during the 12-month follow-up, and the degree of satisfaction in respect of the medical treatment was significantly higher in the 30 kcal group than in the 25 kcal group at 1 year after discharge.

**Conclusions:**

It is considered to be preferable for the caloric intake to be set at 30kcal/kg IBW/day rather than at 25 kcal/kg IBW/day for obese or overweight patients with type 2 diabetes.

## Introduction

Medical nutrition therapy (MNT) is crucial for treating patients with diabetes and preventing the complications of this disease. Therefore, it is important at all levels of treatment of diabetes [1]. The effectiveness of MNT for diabetes patients has been reported by many studies [2-4]. Clinical trials of MNT have demonstrated 1-2% decreases of the HbA1c in patients with type 2 diabetes, depending on the duration of diabetes [3, 5]. As obesity is associated with insulin resistance, decrease of the body weight is very important for individuals with pre-diabetes or diabetes [6].

The concept that MNT should cover a well-balanced healthy diet for diabetic patients is well-recognized around the world. However, the content of MNT in Japan is not the same as that in European or American countries. In Europe and America, carbohydrate counting therapy has spread as effective MNT for diabetic patients. Recent research has suggested the potential usefulness of the dietary glycemic index (GI) for reducing postprandial hyperglycemia [7, 8] and the subsequent insulin demand [9, 10]. In other recent studies, vegetable-based low-carbohydrate diets were associated with lower all-cause and cardiovascular disease mortality rates, and low-carbohydrate diets were associated with favorable changes in the risk factors for cardiovascular diseases [11, 12]. The American Diabetes Association (ADA) suggested that the caloric intake of overweight patients (BMI ≥ 25 kg/m^2^) and obese patients (BMI ≥ 30 kg/m^2^) must be 500 - 1,000 lower than that necessary for weight maintenance [6]. According to the recommendations of the European Association for the Study of Diabetes (EASD), the caloric intake should be reduced and the energy expenditure increased for those who are overweight (BMI ≥ 25 kg/m^2^), so that the BMI moves towards the recommended range (BMI, 18.5 - 25 kg/m^2^) [13]. However, a precise energy setting has not been reported. By contrast, in Japan, the main focus is on energy control, which is somehow different from the case in Europe and America. According to the recommendations of the Japan Diabetes Society (JDS), Japan Atherosclerosis Society (JAS), and Japan Society for the Study of Obesity (JASSO), the ideal body weight (IBW) is calculated as IBW = height (m) ^2^ × 22 (kg/m^2^) [14-16], and the total dietary energy (kcal/day) is estimated as 25 - 35 kcal/kg IBW/day, which depends on the daily activity [14, 16]: subjects with low-level activity, 25 - 30 kcal/kg IBW; subjects with usual-level activity, 30 - 35 kcal/kg IBW; subjects with heavy-level activity, 35 or more kcal/kg IBW; notably, in patients with obesity: 20 - 25 kcal/kg IBW. Finally, physicians are advised to determine the total dietary caloric intake taking into consideration various patient background factors.

Nakajima et al reported that the dietary program recommended by JDS and JASSO is practically useful for body weight control and for improving lipid metabolism in patients with type 2 diabetes [17]. Of note, the total dietary energy intake is often set at 25 kcal/kg IBW/day when MNT is prescribed for obese patients with type 2 diabetes in Japan. A study of the Japan Diabetes Complication Study (JDCS) group [18] reported that average energy intake in Japanese type 2 diabetes patients was 1,700 kcal/day, and that the average BMI of the patients in their investigation was 22.9 ± 3.0 kg/m^2^. It is, however, unknown how many meals overweight diabetic patients with BMI values of more than 25 eat.

Specialists in diabetes may agree with the notion that it is difficult for overweight subjects in modern society to comply with MNT (less than 25 kcal /kg IBW/day). Thus, it may be preferable to set an adequate caloric intake that can actually be complied with rather than to set an ideal, but inadequate energy intake that is impossible to comply with. However, the most appropriate energy intake has not been determined so far. Therefore, we designed a prospective, randomized, controlled, multicenter study trial to determine the impact of the total dietary energy intake (25 or 30 kcal/IBW/day) on the body weight, glycemic control, lipid profile, and satisfaction level in obese or overweight patients with type 2 diabetes mellitus.

## Materials and Methods

### Design overview

We conducted an interview and performed a prospective, randomized, controlled study in Yokohama City University Hospital and Yokohama City University Medical Center in Kanagawa Prefecture. Recruitment of doctors for interview and hospitalization of patients began from September 2008 and continued until June 2010. We had obtained the approval of the Institutional Ethics Review Committee of Yokohama City University Hospital for the conduct of this study, and obtained informed consent from each of the subjects prior to the start of this study (UMIN000002179).

### Interview

We interviewed physicians working at hospitals in Kanagawa Prefecture to clarify the actual status of MNT prescribed by them for obese and overweight type 2 diabetics.

### Setting and participants

The subjects were obese or overweight patients with type 2 diabetes (BMI ≥ 25) who were hospitalized at the Yokohama City University Hospital or Yokohama City University Medical Center at the time of the start of this study.

### Randomization and interventions

Subjects were randomly assigned to the 25 kcal/kg IBW/day group (25 kcal group) or the 30 kcal/kg IBW/day group (30 kcal group).

### Outcomes and follow-up

The primary endpoint was the body weight of the subjects at the time of hospitalization, at the time of discharge from the hospital, and at 3, 6 and 12 months after discharge from the hospital. The secondary endpoints were the degree of glycemic control (HbA1c), the lipid profile (serum LDL-C, HDL-C and triglyceride (TG)), continuation and performance of dietary treatment (energy intake), level of satisfaction and any psychological complaints (questionnaire survey).

Glycemic control: The serum HbA1c level was evaluated at the time of hospitalization and at the 3, 6 and 12 months after discharge from the hospital. Lipid profile: Serum levels of LDL-C, HDL-C and TG were evaluated at the time of hospitalization, at the time of discharge from the hospital, and at 3, 6 and 12 months after discharge from the hospital. The HbA1c (%) value was estimated as the National Glycohemoglobin Standardization Program (NGSP) equivalent value (%) calculated using the formula: HbA1c (%) = HbA1c (JDS) (%) + 0.4%, considering the relational expression of HbA1c (JDS) (%) measured by the previous Japanese standard substance and measurement method and HbA1c (NGSP) [19]. The meal calorie interview was conducted to estimate the actual amount of meals that the subjects ate before hospitalization and at 3, 6 and 12 months after discharge from the hospital.

Moreover, a questionnaire survey was conducted before the subjects’ discharge from the hospital and at 6 and 12 months after discharge from the hospital to evaluate the degree of satisfaction with their diabetes treatment regimen (Diabetes Treatment Satisfaction Questionnaire (DTSQ) [20]) and the degree of burden (Problem Areas in Diabetes Survey (PAID) [21]). The DTSQ consists of 8 items, each with 7 possible answers. Six of the eight items were designed specifically to measure a person’s level of satisfaction with his/her diabetes treatment regimen (‘current treatment’, ‘convenience’, ‘flexibility’, ‘understanding’, ‘recommend’ and ‘continue’) [20]. Two additional items concerned with the perceived frequency of hypo- and hyperglycemia were scored separately as single items. The individual questions on the DTSQ were scored on a 7-point Likert scale, from 0 to 6. In the present study, the total score for the six items was used as the treatment satisfaction scale. The total scores obtained by summing these six items from the DTSQ ranges from 0 to 36. In the present study, the Japanese version of the DTSQ, which was linguistically and psychometrically evaluated by Ishii et al [22], was used. The PAID is a measure of diabetes-specific emotional distress, that was developed by Polonsky et al [23] and translated into Japanese by Ishii et al [24]. The scale consists of 20 items with 5 response options available for each PAID question: each response option was assigned a value of 0 (not a problem) to 4 (serious problem). According to the recommendation of the measure’s authors, a total score was computed by summing the responses and multiplying this total by 1.25 to obtain a total score that ranges from 0 - 100.

### Statistical analysis

Statistical analyses were performed using the SPSS for Windows, Japanese version 16.0 (SPSS Institute Inc., Japan). Statistical comparisons of the temporal changes in each group of each item and of the changes in the body weight, amount of actually ingested energy and laboratory data between the 25 and 30 kcal groups were studied by Bonferroni’s multiple comparison. Comparisons of the DTSQ and PAID scores between the 25 and 30 kcal groups were performed using the Wilcoxon’s rank-sum test. Comparisons of the change of the attendance rate between the 25 and 30 kcal groups were performed using the mantel-Haenszel test. All the data were expressed as mean ± S.D. P values < 0.05 were considered to denote statistical significance. Data were expressed as mean ± SD.

## Results

### Interview

Of the 82 physicians interviewed by us, 77 reported prescribing an energy intake for obese and overweight (BMI ≥ 25) patients with type 2 diabetes. The prescriptions were as follows: less than 25 kcal/kg IBW/day, 18.2%; 25 - 30 kcal/kg IBW/day, 76.6%; 30kcal/kg IBW/day or more, 5.2%. In regard to whether the physicians believed that their patients would be able to comply with their energy prescriptions, the interview revealed the following: Yes, 13.5%; Not sure, 19.1%; No, 67.4%. The compliance rate with the prescribed energy intake if the physicians themselves were diabetic was as follows; less than 25 kcal/kg IBW/day, 6.3%; 25 - 30 kcal/kg IBW/day, 54.4%; 30 kcal/kg IBW/day or more, 39.2%.

### Profiles of the patients

**Figure 1:**
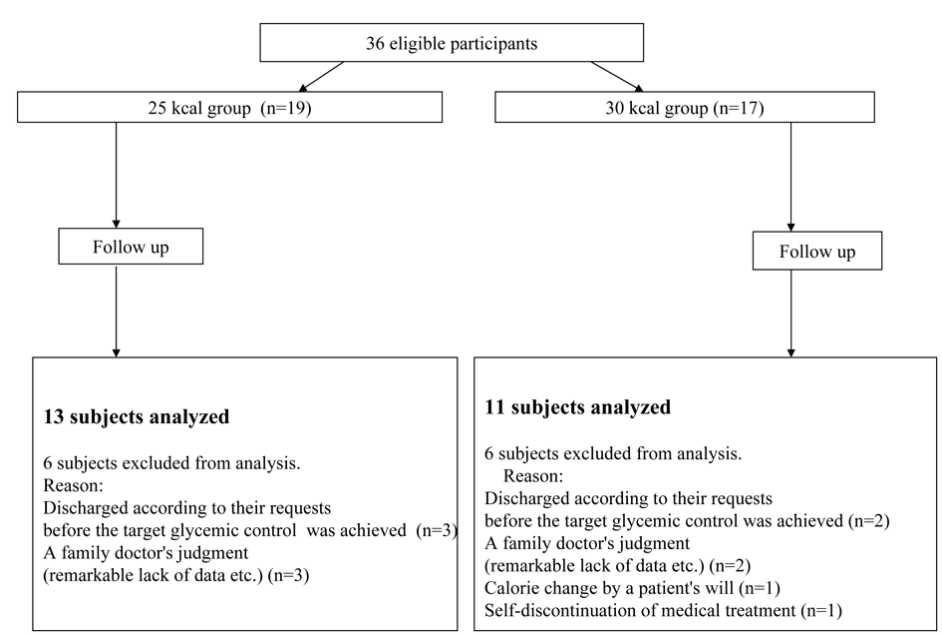
Flow-diagram of the study. The diagram shows the number of patients followed up at different times during the study period.

Of the 36 subjects enrolled in the study, 19 patients were assigned to the 25 kcal group and 17 patients to the 30 kcal group (Fig. 1). Of these, 6 subjects of the 25 kcal group and 6 of the 30 kcal group dropped out of the study before the end of the 1-year study period. Therefore, the remaining 13 subjects of the 25 kcal group and 11 subjects of the 30 kcal group were included in the analysis of the present study. No significant differences in the demographic or clinical parameters were observed between the two groups at the baseline (Table 1).

**Table 1:** Profiles of Patients of the Two Groups at Baseline

	25 kcal group	30 kcal group	P value
Gender (M/F)	10/3	6/5	0.24
Age (years)	58.8 ± 12.1	61.5 ± 12.3	0.61
Duration of diabetes (years)	10.7 ± 9.53	8.23 ± 7.41	0.49
BMI (kg/m^2^)	27.9 ± 2.35	30.1 ± 3.90	0.11
HbA1c (%)	8.88 ± 1.01	9.43 ± 1.62	0.33
Estimation of the amount of energy intake (kcal/day)	2,182 ± 822.3	2,082 ± 540.5	0.73

Data are expresses as mean ± SD.

### Changes in the BMI

**Figure 2:**
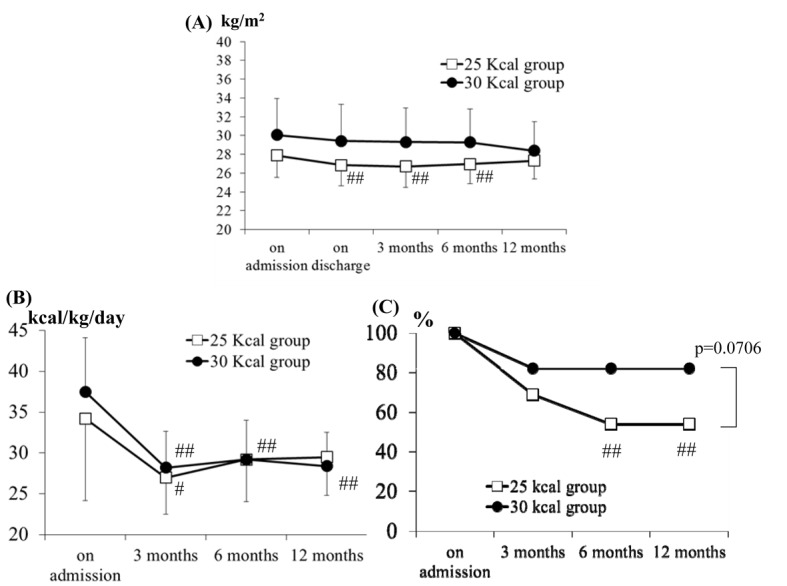
Time-profiles of the BMI, the actual energy intake (per IBW) and the attendance rate. A: Time-profiles of the BMI in the 25 and 30 kcal groups. There were no significant differences at any time-point between the 25 and 30 kcal groups. The data shown are the mean ± SD. ^##^P < 0.01 vs. baseline. B: Time-profiles of the actual energy intake (per IBW). There were no significant differences at any time-point between the 25 and 30 kcal groups. The data shown are the mean ± SD. ^#^P < 0.05, ^##^P < 0.01 vs. baseline. C: Time-profiles of the attendance rate. There were no significant differences in the rate at each time-point between the 25 and 30 kcal groups. ^##^P < 0.01 vs. hospitalized (Bonferroni’s multiple comparison). P value: 25 kcal group vs. 30 kcal group (Mantel-Haenszel test).

There were no significant differences in the BMI values at any of the time-points between the 25 and 30 kcal groups. As shown in Figure 2A, the BMI values at discharge, and at 3 and 6 months after discharge were significantly lower than the values before hospitalization in the 25 kcal group. The BMI tended to decrease from the time of hospitalization to 12 months after discharge from the hospital in the 30 kcal group, however, the difference was not statistically significant.

### Results of interview in respect of the caloric intake and the status of nutrition instruction attendance

There were no significant differences in the actual energy intake (per IBW) at any time-point between the 25 and 30 kcal groups. In the 25 kcal group, the actual energy intake per IBW was significantly lower (P = 0.019) only at 3 months after discharge, as compared with that before hospitalization. In the 30 kcal group, on the other hand, the actual energy intake per IBW was significantly lower at 3 months (P < 0.01), 6 months (P < 0.01) and 12 months (P < 0.01) after discharge as compared with that before hospitalization. It is noteworthy that the actual energy intake per IBW at 12 months after discharge was approximately 30 kcal/kg/day in the 25 kcal group, and approximately 27 kcal/kg/day in the 30 kcal group (Fig. 2B).

The nutrition instructions were provided to the patients by the physician in charge at the time of each visit. Although the instructions were provided at the time of hospitalization to all patients (100%), 69, 54 and 54% of the 25 kcal group, and 82, 82 and 82% of the 30 kcal group also received instructions subsequently at 3, 6 and 12 months after discharge, respectively (Fig. 2C). Thus, the attendance rate in the 25 kcal group tended to be lower than that in the 30 kcal group. The attendance rates in the 25 kcal group were significantly lower at 6 and 12 months after discharge (P < 0.01) than the rate at the time of hospitalization. The reasons for the reduced compliance with the nutrition instruction by the enrolled subjects were as follows; “I have no time”, “I have understood it well”, “the real caloric intake is already within the limit of instruction”, “it is meaningless for me”, and “it is stressful for me to attend the nutrition lecture”, etc.

### Changes in the serum HbA1c, LDL-C, HDL-C and TG

**Figure 3:**
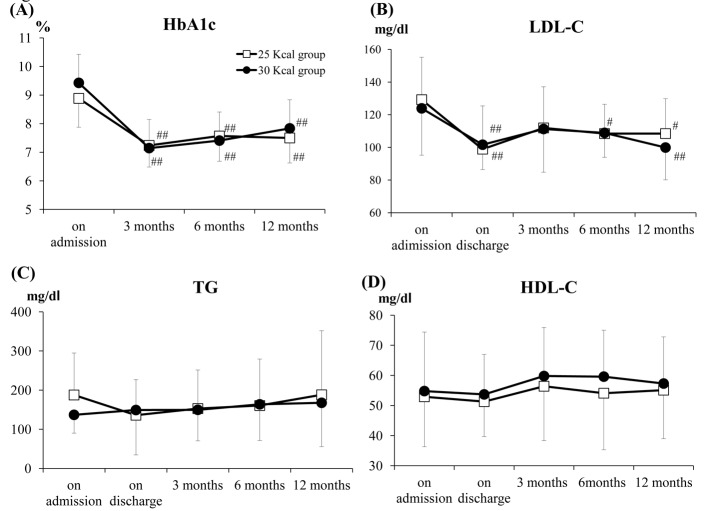
Time-profiles of the HbA1c (NGSP) levels and lipid parameters. A: Time-profiles of the plasma HbA1c (NGSP) levels. B: Time-profiles of the plasma LDL cholesterol levels. C: Time-profiles of the plasma TG levels. D: Time profiles of the plasma HDL cholesterol levels. There were no significant differences at any of the time-points between the 25 and 30 kcal groups. The data shown are the mean ± SD. ^#^P < 0.05, ^##^P < 0.01 vs. baseline.

Significant decreases of the HbA1c values were observed at 3 months (P < 0.01), 6 months (P < 0.01) and 12 months (P < 0.01) after discharge from the hospital in both the 25 and 30 kcal groups as compared with the values before hospitalization (Fig. 3A). In regard to the lipid profile, significant decreases of the serum LDL-C levels as compared with the level before hospitalization were observed at discharge (P < 0.01), and at 6 months (P = 0.0126) and 12 months (P = 0.0126) after discharge from the hospital in the 25 kcal group. On the other hand, in the 30 kcal group, significant decreases of the serum LDL-C levels as compared with the baseline value were observed at discharge (P < 0.01) and at 12 months (P < 0.01) after discharge from the hospital (Fig. 3B). There were no significant differences in the serum levels of HDL-C or TG at 3, 6 and 12 months after discharge from the hospital as compared with the levels at the baseline in either group (Fig. 3C). Collectively speaking, there were no significant differences in the changes in the serum HbA1c, LDL-C, HDL-C or TG at each time-point between the 25 and 30 kcal groups.

### The scores on the DTSQ and PAID

**Figure 4:**
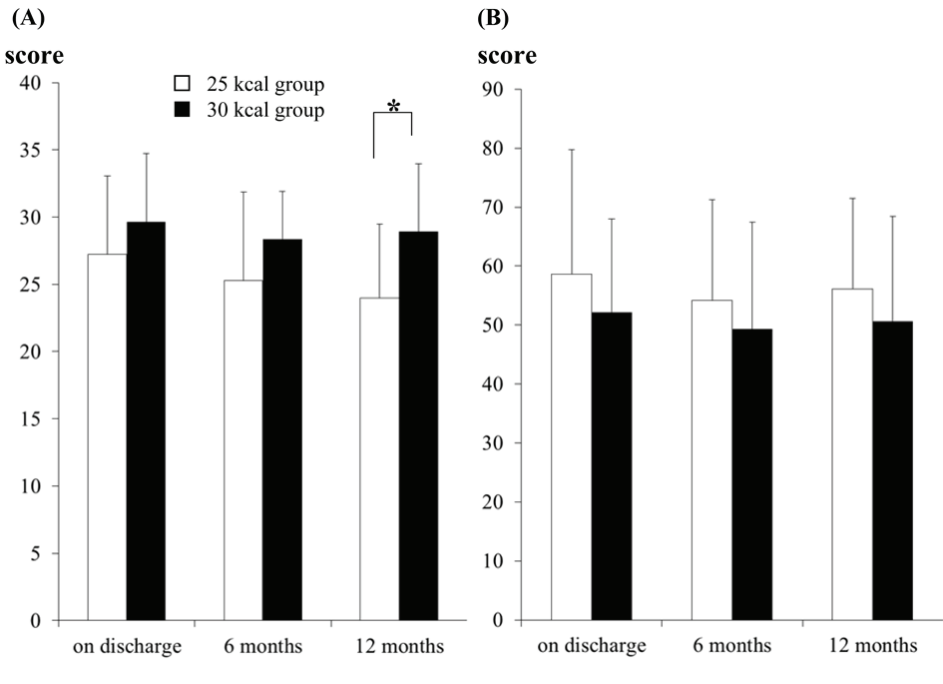
The total score for the six items of the Diabetes Treatment Satisfaction Questionnaire (DTSQ) and Problem Areas in Diabetes Survey (PAID). A: The total score for the six items on the DTSQ in the 25 kcal group (open bars) and 30 kcal group (filled bars). The data are shown as the mean ± SD. At 12 months after discharge, significant difference was seen between the two groups (*P < 0.05). B: The total PAID scores in the 25 kcal group (open bars) and 30 kcal group (filled bars). The data shown are the mean ± SD in the 25 and 30 kcal groups. n.s. indicates not significant.

The DTSQ score (the total score for the six items) at 12 months after discharge from the hospital was significantly higher in the 30 kcal group than in the 25 kcal group (Fig. 4A). Although there was no significant difference in the PAID scores at any of the time-points between the two groups, the scores in the 30 kcal group always tended to be lower than those in the 25 kcal group (Fig. 4B).

## Discussion

The most important findings of our study were the absence of any significant differences in the glycemic control, improvement of lipid profile, or body weight change between the 25 and 30 kcal groups after 12 months, and the significantly higher degree of satisfaction with the prescribed medical treatment regimen in the 30 kcal group than in the 25 kcal group at 12 month after discharge.

Unlike in the ADA [6] and EASD [13], the main focus of MNT is on energy control in Japan [14-16]. It is common in MNT in Japan to calculate a patient’s IBW before prescribing the daily energy intake in all subjects, including obese subjects. In this process of diabetes care, physicians have become accustomed to setting the energy intake at 25 or less kcal/kg IBW/day for overweight diabetic subjects, with the goal of reducing the body weight. In the clinical study mentioned above [17], the dietary caloric intake was set at 25 kcal/kg IBW/day, irrespective of the BMI. These diet instructions provided appropriate caloric restriction depending on the BMI and induced reasonable body weight reduction in both obese and normal-weight subjects. They reported that the dietary program recommended by the JDS, JAS and JASSO is practically useful for body weight control in type 2 diabetic patients [17]. However, this study only focused on the short-term effectiveness of MNT for patients who were hospitalized.

In this study, there were no significant differences in the glycemic control, improvement of lipid profile or body weight change between the 25 and 30 kcal groups after 12 months, and the actual dietary intake was significantly improved at 12 months after discharge from the hospital, as compared with that at the baseline, in the 30 kcal /kg/day group. Because obese or overweight patients are probably accustomed to a higher daily intake of calories as compared to normal-weight subjects, it is difficult for them to comply with MNT over the long term if a very low daily energy intake is prescribed (25 kcal/kg IBW/day). Wing et al also reported on year-long weight loss treatment for obese patients with type 2 diabetes [25]. The subjects in the low calorie diet group of 1,000 to 1,200 kcal/day achieved their maximum weight loss at 6 months (13.5 kg loss) and then regained weight (2.1 kg gain) after 6 to 12 months. This result raised a major concern regarding the sustainability of MNT in obese patients with type 2 diabetes. While the BMI showed a gentle upward trend at 12 months in the 25 kcal group, it still showed a downward trend in the 30 kcal group (Fig. 2A). The final average of BMI in the 25 and 30 kcal groups may approach each other in the long-term.

Hanefeld et al evaluated the efficacy of intensified health education (IHE) in improving metabolic control in a randomized 5 year multi-intervention trial [26]. For the IHE group, oral and written instructions were given for low-calorie diets of about 800 - 1,500 kcal/day. It was found that after 5 years, the IHE had no effect on the caloric intake, percentage of fat in the diet, or the body weight. Consistent with their study, the 25 kcal group actually showed a tendency towards a lower nutrition instruction attendance rate as compared with the 30 kcal group at 6 and 12 months after discharge from hospital (P = 0.0706) (Fig. 2C). More importantly, the DTSQ score was significantly higher in the 30 kcal group than in the 25 kcal group. Therefore, it is preferable to prescribe a more practical caloric intake that would provide higher satisfaction and is easy to sustain than excessively restrict caloric intake, for example, 25 kcal/kg IBW/day, to improve the long-term adherence with MNT.

The results of the questionnaire survey of physicians about MNT revealed that many physicians prescribed a caloric intake in the range of 25 - 30 kcal/kg IBW/day, although they did not expect their patients to actually comply with the instructions. Moreover, they themselves thought that it was difficult to comply with the caloric restriction (25 - 30 kcal/kg IBW/day). The results of the questionnaire suggested that the MNT prescribed by physicians in Japan has not been effective for obese patients with diabetes.

There were several limitations to this study. As the number of subjects in this study was small and the term of the study was short, larger-scale and longer-term studies in the future would be warranted.

In conclusion, although there were no significant differences in the degree of glycemic control, improvement of the serum LDL-C, or body weight change between the 25 kcal/kg/day and 30 kcal/kg/day groups at 12 months after discharge from hospital, the degree of satisfaction with the prescribed medical treatment at 12 months after leaving hospital was significantly higher in the 30 kcal group than in the 25 kcal group. Therefore, it is considered to be preferable for obese patients with type 2 diabetes to be prescribed 30 kcal/kg IBW/day than 25 kcal/kg IBW/day. Our results are expected to be helpful to decide a more practical daily caloric intake for overweight and obese patients with type 2 diabetes.

## References

[1] Bantle JP, Wylie-Rosett J, Albright AL, Apovian CM, Clark NG, Franz MJ, Hoogwerf BJ (2008). Nutrition recommendations and interventions for diabetes: a position statement of the American Diabetes Association. Diabetes Care.

[2] (1990). UK Prospective Diabetes Study 7: response of fasting plasma glucose to diet therapy in newly presenting type II diabetic patients, UKPDS Group. Metabolism.

[3] Pastors JG, Warshaw H, Daly A, Franz M, Kulkarni K (2002). The evidence for the effectiveness of medical nutrition therapy in diabetes management. Diabetes Care.

[4] Wing RR, Blair EH, Bononi P, Marcus MD, Watanabe R, Bergman RN (1994). Caloric restriction per se is a significant factor in improvements in glycemic control and insulin sensitivity during weight loss in obese NIDDM patients. Diabetes Care.

[5] Pastors JG, Franz MJ, Warshaw H, Daly A, Arnold MS (2003). How effective is medical nutrition therapy in diabetes care?. J Am Diet Assoc.

[6] Klein S, Sheard NF, Pi-Sunyer X, Daly A, Wylie-Rosett J, Kulkarni K, Clark NG (2004). Weight management through lifestyle modification for the prevention and management of type 2 diabetes: rationale and strategies: a statement of the American Diabetes Association, the North American Association for the Study of Obesity, and the American Society for Clinical Nutrition. Diabetes Care.

[7] Brand-Miller J, Hayne S, Petocz P, Colagiuri S (2003). Low-glycemic index diets in the management of diabetes: a meta-analysis of randomized controlled trials. Diabetes Care.

[8] Ludwig DS (2002). The glycemic index: physiological mechanisms relating to obesity, diabetes, and cardiovascular disease. JAMA.

[9] Higgins JA, Brand Miller JC, Denyer GS (1996). Development of insulin resistance in the rat is dependent on the rate of glucose absorption from the diet. J Nutr.

[10] Byrnes SE, Miller JC, Denyer GS (1995). Amylopectin starch promotes the development of insulin resistance in rats. J Nutr.

[11] Foster GD, Wyatt HR, Hill JO, Makris AP, Rosenbaum DL, Brill C, Stein RI (2010). Weight and metabolic outcomes after 2 years on a low-carbohydrate versus low-fat diet: a randomized trial. Ann Intern Med.

[12] Fung TT, van Dam RM, Hankinson SE, Stampfer M, Willett WC, Hu FB (2010). Low-carbohydrate diets and all-cause and cause-specific mortality: two cohort studies. Ann Intern Med.

[13] Mann JI, De Leeuw I, Hermansen K, Karamanos B, Karlstrom B, Katsilambros N, Riccardi G (2004). Evidence-based nutritional approaches to the treatment and prevention of diabetes mellitus. Nutr Metab Cardiovasc Dis.

[14] (2012). Japan Diabetes Society: Guideline For Treatment Of Diabetes Mellitus 2012-2013. Bunkyo-do, Tokyo.

[15] (2012). Japan Atherosclerosis Society: Japan Atherosclerosis Society (JAS) Guidelines for Prevention of Atherosclerosis Cardiovascular Diseases.

[16] Teramoto T, Sasaki J, Ueshima H, Egusa G, Kinoshita M, Shimamoto K, Daida H (2008). Treatment - therapeutic lifestyle modification. J Atheroscler Thromb.

[17] Nakajima Y, Sato K, Sudo M, Nagao M, Kano T, Harada T, Ishizaki A (2010). Practical dietary calorie management, body weight control and energy expenditure of diabetic patients in short-term hospitalization. J Atheroscler Thromb.

[18] http://medical.nikkeibp.co.jp/leaf/all/gakkai/jds2011/201105/519835.html.

[19] (2010). The Committee of Japan Diabetes Society on the Diagnostic Criteria of Diabetes Mellitus. Report of the Committee on the classification and diagnostic criteria of diabetes mellitus. J Diabetes Invest.

[20] Bradley C, Bradley C (1994). Diabetes treatment satisfaction questionnaire (DTSQ). Handbook of Psychology and Diabetes: A guide to psychological measurement in diabetes research and practice.

[21] Snoek FJ, Pouwer F, Welch GW, Polonsky WH (2000). Diabetes-related emotional distress in Dutch and U.S. diabetic patients: cross-cultural validity of the problem areas in diabetes scale. Diabetes Care.

[22] Ishii H, Bradley C, Riazi A, Barendse S, Yamamoto T (2000). The Japanese version of the Diabetes Treatment Satisfaction Questionnaire (DTSQ): translation and clinical evaluation. J Clin Exp Med.

[23] Polonsky WH, Anderson BJ, Lohrer PA, Welch G, Jacobson AM, Aponte JE, Schwartz CE (1995). Assessment of diabetes-related distress. Diabetes Care.

[24] Ishii H (2000). Psycho-behavioral problems in diabetes treatment. J Jpn Diab Soc.

[25] Wing RR, Blair E, Marcus M, Epstein LH, Harvey J (1994). Year-long weight loss treatment for obese patients with type II diabetes: does including an intermittent very-low-calorie diet improve outcome?. Am J Med.

[26] Hanefeld M, Fischer S, Schmechel H, Rothe G, Schulze J, Dude H, Schwanebeck U (1991). Diabetes Intervention Study. Multi-intervention trial in newly diagnosed NIDDM. Diabetes Care.

